# Human adipose‐derived multipotent stromal cells enriched with IL‐10 modRNA improve diabetic wound healing: Trigger the macrophage phenotype shift

**DOI:** 10.1002/btm2.10711

**Published:** 2024-08-07

**Authors:** Yuxin Zhang, Wei Wang, Liang Chen, Heng Wang, Dong Dong, Jingjing Zhu, Yu Guo, Yiqun Zhou, Tianyi Liu, Wei Fu

**Affiliations:** ^1^ Shanghai Key Laboratory of Clinical Geriatric Medicine Huadong Hospital Shanghai China; ^2^ Department of Plastic Surgery, Huadong Hospital, School of Medicine Fudan University Shanghai China; ^3^ Institute of Pediatric Translational Medicine, Shanghai Institute of Pediatric Congenital Heart Disease, Shanghai Children's Medical Center, School of Medicine Shanghai Jiao Tong University Shanghai China

**Keywords:** adipose‐derived multipotent stromal cells, diabetic wound, interleukin‐10, stem cell therapy, therapeutic mRNA

## Abstract

Diabetic wounds present a significant challenge in regenerative medicine due to impaired healing, characterized by prolonged inflammation and deficient tissue repair, primarily caused by a skewed pro‐inflammatory macrophage phenotype. This study investigates the therapeutic potential of interleukin‐10 (IL‐10) chemically modified mRNA (modRNA)‐enriched human adipose‐derived multipotent stromal cells (hADSCs) in a well‐established murine model of diabetic wounds. The modRNAs used in this study were chemically modified using N1‐methylpseudouridine‐5′‐triphosphate (m1Ψ) by substituting uridine‐5‐triphosphate. In vitro experiments demonstrated that IL‐10 modRNA‐transfected hADSCs effectively modulated macrophage polarization towards an anti‐inflammatory phenotype. In vivo experiments with a well‐established murine model demonstrated that transplantation of hADSCs^modIL‐10^ on postoperative day 5 (POD5) significantly improved wound healing outcomes, including accelerated wound closure, enhanced re‐epithelialization, promoted M2 polarization, improved collagen deposition, and increased neovascularization. This study concludes that IL‐10 modRNA‐enriched hADSCs offer a promising therapeutic approach for diabetic wound healing, with the timing of IL‐10 administration playing a crucial role in its effectiveness. These cells modulate macrophage polarization and promote tissue repair, demonstrating their potential for improving the management of diabetic wounds.

**Translational Impact Statement**

By utilizing adipose‐derived multipotent stromal cells enriched with chemically modified IL‐10 mRNA, this study presents a promising therapeutic approach capable of inducing a shift in macrophage phenotype, leading to improved wound closure and tissue repair. The findings from this study hold substantial translational implications for the advancement of innovative stem cell‐based therapies intended to enhance healing outcomes for individuals suffering from diabetic wounds.

## 1 INTRODUCTION

Aberrant or delayed wound healing process, such as diabetic foot ulcers (DFUs), presents a significant challenge in the management of diabetes, affecting millions of individuals worldwide. According to the latest edition of *IDF Diabetes Atlas* issued by the *International Diabetes Federation*,^1^ over 537 million people are living with diabetes mellitus (DM) today and the number will reach 700 million by 2045 as estimated. The diabetes‐related health expenditure in 2021 has reached over 966 billion USD and is expected to reach 1054 billion USD by 2045. DFU is the first‐line cause of lower extremity amputation (around 67%) in the United States.^2^ Meanwhile, DFU is one of the most expensive chronic wounds to treat and has a comparable 5‐year mortality rate to cancer (30.5% vs 31%) but the research depth of it is far less than the oncology field.^3^ Collectively, diabetes‐related chronic wound, calls for an urgent need for comprehensive prevention and management strategy.

Multifactorial abnormalities contribute to impaired diabetic wound healing, including persistent inflammation, oxidative stress, angiogenesis dysfunction, and impaired extracellular matrix remodeling.^4^ Macrophages, key immune cells involved in the wound‐healing process, play pivotal roles in orchestrating various stages of tissue repair and regeneration by altering their phenotypes in response to environmental cues.^5^ Manipulation of macrophage phenotype has emerged as a promising therapeutic approach in various diseases and conditions including diabetic wounds.^6^, ^7^, ^8^, ^9^ Generally, the pro‐inflammatory M1 macrophage assumes dominance (85%) from the initial stages of wound formation until the 3rd day. Subsequently, a transition takes place, leading to the prevalence of anti‐inflammatory M2 macrophages (80%–85%) from day 5 to day 7.^4^, ^10^ In the context of diabetic chronic wounds, the phenotypic switch of macrophages into repair mode is disturbed.^11^ Previous research findings indicate that significant differences in cytokines and growth factors between macrophages in diabetic and non‐diabetic wounds start to emerge on the 10th day, while they exhibit similar levels on the 5th day.^12^ Interleukin‐10 (IL‐10) is a classic anti‐inflammatory cytokine secreted by activated immune cells to regulate inflammation.^13^ The research conducted by Ip et al reveals that IL‐10 plays a major role in inflammation resolution by controlling cellular metabolism of macrophages via inhibiting mTORC1.^14^ Moreover, IL‐10 dysregulation was shown to impair wound healing in diabetic wounds via chemokine insufficiency and delayed macrophage response.^15^ Collectively, IL‐10 was considered a promising therapeutic candidate for regulating the skewed inflammatory microenvironment in diabetic wounds. However, the clinical application of IL‐10 in diabetic wound management is still under investigation.

Numerous studies reported the use of IL‐10‐engineered multipotent stromal cells (MSC) in various inflammation‐related diseases.^16^, ^17^, ^18^, ^19^ MSCs serve as carriers to achieve efficient expression of IL‐10 within the local tissues, thereby exerting corresponding therapeutic effects. Compared to other MSCs, adipose‐derived multipotent stromal cells are more easily accessible and can be obtained from autologous or allogeneic adipose tissues in the abdominal region, inguinal region, and thighs during liposuction procedures.^20^ The therapeutic potential of ADSCs in diabetic wound healing has been supported by numerous preclinical and clinical studies.^21^ Notably, the latest viewpoint suggests that transplanted ADSCs do not retain and replace the local tissue but instead exert a ‘hit‐and‐run’ effect.^22^, ^23^ Current research focuses on enhancing the expression of therapeutic proteins in ADSCs through modifications to foster their effects post‐transplantation.^24^, ^25^ However, considering the off‐target effects and risk of genomic integration associated with viral vectors, most clinical trials still employ the primary ADSCs.^21^, ^26^ The relatively low clinical translation rate of ADSC therapy underscores the need for further research to develop safer and more efficient methods to modify ADSCs, specifically to enhance the expression of therapeutic proteins. The latest studies on MSCs modifications technique used in diabetic wounds include enhancing VEGFA expression in bone marrow multipotent stromal cells (BM‐MSCs) through the electroporated‐Cas9‐AAV6 platform and enhancing hepatocyte growth factor (HGF) and C‐X‐C motif chemokine ligand 12 (CXCL12) expression in ADSCs through the LNP‐mRNA/saRNA platform.^26^, ^27^ Recent studies have supported that the chemically modified mRNA technique allows for transient, localized, and intense expressions of therapeutic proteins in transfected cells, enabling a therapeutic effect.^28^, ^29^, ^30^, ^31^ The integrations of ADSC therapy with chemically modified mRNAs (modRNA) have demonstrated therapeutic efficacy in diverse diseases and conditions, including limb ischemia,^32^ bone defects,^30^ and fat grafting.^28^ Compared with traditional lentivirus vectors or other stem cell gene therapies, modRNA technology shows a better safety profile, allowing transient expression of therapeutic proteins without being integrated into the genome. This reduces the risk of insertional mutagenesis and off‐target effects.^33^, ^34^ In our previous research, we highlighted the synergistic potential of TGF‐β3 and IL‐10 modRNA‐loaded hADSCs in establishing a scar‐free wound‐healing microenvironment in a dorsal injury rat model for managing wounds prone to scar formation.^35^ These modRNA‐modified hADSCs exhibited multidimensional therapeutic effects, including improved collagen deposition, extracellular matrix organization, neovascularization, and inhibition of keloid fibroblast proliferation and migration. Taken together, we proposed that IL‐10 modRNA‐enriched ADSCs therapy might be beneficial in enhancing the inflammation resolution potential of ADSCs and the gain‐of‐function trait of modRNA could trigger the macrophage polarization more precisely. Our current study specifically investigates the promotive effects of hADSCs as carriers for delivering IL‐10 modRNA on the healing process of diabetic chronic wounds and its potential mechanisms. We emphasize the impact of hADSCs^modIL‐10^ on macrophage polarization and related healing. Additionally, we have included preliminary discussions on the timing of IL‐10 administration. As a conceptual exploration, the main goal of this research was to evaluate the effects of IL‐10 modRNA‐transfected ADSCs in enhancing diabetic wound healing, regulating macrophage phenotype, and thus providing a comprehensive scheme for diabetic wound management.

## 2 MATERIALS AND METHODS

### 2.1 Cell isolation, Culture, and Characterization

The adipose tissues used in this study were derived from five healthy female patients who underwent liposuction procedures in the abdominal region with ages ranging from 25 to 35 years old. Informed Consent Forms were signed by patients before surgery. Based on the established protocols,^36^ lipoaspirates were rinsed with PBS, centrifugated, and aspirated firstly, and then digested with 0.05% collagenase type I at 37°C for 1 h. The mixture was then filtrated, and the liquid part was then centrifugated at 400 g for 10 min. The lower portion majorly containing intact adipocytes and preadipocytes was centrifugated again at 400 g for 10 min. Then the cell pellet was resuspended in Dulbecco's modified Eagle's medium (DMEM) (Thermo Fisher Scientific, C11995500BT) supplemented with 20% fetal bovine serum (FBS) (Beyotime, C0232) and 1% penicillin–streptomycin solutions (ABCONE, P33067, 100 μg/mL) and seeded onto the dishes. The cells were cultured at 37°C with 5% CO2. The cells used in this study were between passages 3 and passage 6. The macrophage cell line RAW264.7 was obtained from the Stem Cell Bank, Chinese Academy of Sciences. RAW264.7 cells were cultured using Dulbecco's modified Eagle's medium (DMEM) (Thermo Fisher Scientific, C11995500BT) supplemented with 10% fetal bovine serum (FBS) (Beyotime, C0232) and penicillin–streptomycin (ABCONE, P33067, 100 μg/mL). To characterize the hADSCs, we performed flow cytometry analysis to assess surface marker expression. Flow cytometry was used to verify the expression of surface markers CD73, CD90, CD105, and CD45 on hADSCs (Figure S1).

### 2.2 ModRNA Synthesis and Formulation

The synthesis of chemically modified mRNA was performed using an in vitro process. Initially, a linearized DNA template containing generic 5′ and 3′ UTRs (untranslated regions) and a poly‐A tail were utilized, following a previously described method.^37^ In the in vitro transcription reaction, uridine was fully substituted with N1‐methylpseudouridine. To purify the synthesized RNA, Ambion MEGAclear spin columns were employed. Additionally, Antarctic phosphatase from New England Biolabs was used to remove any residual 5′‐phosphates, and the treatment was carried out for 30 min at 37°C. Subsequently, the RNA underwent another purification step and was quantified using a NanoDrop spectrophotometer from Thermo Scientific. The resulting modified RNA, referred to as modRNA, was resuspended in a solution of 10 mM Tris‐HCl and 1 mM EDTA at a concentration of 1 μg/μL for future use. For the production of modRNA encoding Green Fluorescent Protein (GFP) and IL‐10, the respective open reading frame sequences were provided in the supporting information (Table S1).

### 2.3 ModRNA Transfection

To transfect hADSCs with modRNA, Messenger‐MAX (Invitrogen, California, USA) transfection reagent was employed as the transmitter. ModRNAs and Messenger‐MAX were first diluted separately in Opti‐MEM medium (Invitrogen, California, USA) and incubated for 5 min at room temperature (RT). Afterward, the two mixes were pooled together and incubated for 15 min at RT to generate modRNA‐lipid complexes. The complexes were then exposed to cells for 4–6 h, after which the medium was completely replaced with cell culture media or removed to collect cells for the next procedures. The lipo‐complex containing 5 μL of MessengerMAX transfection reagent with 2 μg modRNA was used to transfect 1 × 10^5^ hADSCs for all following experiments. To assess the expression kinetics of modified mRNA in hADSCs, we detected the GFP signals and the mean fluorescence intensities of modGFP using a confocal scanning laser microscope (Leica) at 4‐, 24‐, 48, and 72 h post‐transfection.

### 2.4 Enzyme‐linked Immunosorbent Assay (ELISA)

To determine the IL‐10 expression kinetics of untreated hADSC, hADSC^modGFP^ and hADSC^modIL‐10^, we collected cell culture supernatants at specific time points (0‐, 4‐, 8‐, 16‐, 24‐, 48‐, and 72‐h after transfection) and quantified the concentration of human IL‐10 protein using ELISA (PeproTech, BGK2301) following the manufacturer's instructions. We measured the optical density values of absorbance using a microplate reader (ELX800, BioTek, USA).

### 2.5 Migration Assay

Migration assay was performed using a 24‐well polycarbonate membrane cell culture plate (Corning). After transfection, hADSCs, hADSCs^modGFP^, and hADSCs^modIL‐10^ were prepared by washing them with PBS, trypsinization, and centrifugation. After that, cell pellets were resuspended in serum‐free medium at a concentration of 5 × 10^4^ cells/mL and seeded on the top chambers of the transwell inserts. The top chambers contained 100 μL cell suspension, and the bottom chambers contained 600 μL of 20% FBS DMEM. After 24 h of incubation in a humidified incubator at 37°C, cells passed through the membrane were fixed with 4% paraformaldehyde for 15–30 min at RT and dyed with crystal violet for 15–30 min at RT. The migrated cells per field were captured under a light microscope and quantified using ImageJ analysis software.

### 2.6 Proliferation Assay

To perform a cell proliferation assay, hADSCs, hADSCs^modGFP^, and hADSCs^modIL‐10^ were seeded in a 96‐well plate with five replicates per group, at a concentration of 2 × 10^3^ cells per well. After transfection, the cells were incubated for a specific period to allow for cell growth. The Cell Counting Kit‐8 (CCK8) (Dojindo, Kumamoto, Japan) was used to measure cell proliferation at different time points following treatment (Day 0, Day 3, Day 5, and Day 7). The optical density was measured at 450 nm by a microplate reader (ELX800, BioTek, USA).

### 2.7 Multipotent Differentiation Experiment

The multipotent properties of hADSCs with or without transfection were tested by inducing their differentiation into multiple cell lineages. According to instructions, pretreated‐hADSCs were cultured separately in OsteoDiff (cyagen Biosciences, HUXMD‐90021) and AdipoDiff Medium (cyagen Biosciences, HUXMD‐90031), followed by Alizarin Red S, and Oil Red O stainings to confirm osteocytes and adipocytes, respectively. Images of differentiated hADSCs were obtained under a light microscope. For the osteogenic differentiation induction experiment, hADSCs were prepared in a six‐well plate at a density of 2 × 10^4^ cells/cm,^2^ with 2 mL of complete culture medium added per well. The cells were then incubated in a CO_2_ incubator at 37°C with 5% CO_2_ and saturated humidity. When the cell confluence reached 70%–80%, the complete culture medium was replaced with Oricel!® OsteoDiff medium in each well. Every 3 days, the medium was further replaced with fresh medium. After 2–4 weeks of induction, morphological changes and growth of the cells were observed, and osteogenic differentiation was assessed using Alizarin Red S staining. For adipogenic differentiation induction, the difference lies in the alternating use of Solution A for 3 days and Solution B for 1 day to induce cell adipogenesis. Both solutions are prepared according to the product manual. Solution A stimulates the formation of lipid droplets, while Solution B maintains the formed lipid droplets and promotes their enlargement. This process of induction and maintenance is repeated until a sufficient amount of appropriately sized lipid droplets is observed, at which point the cells are ready for Oil Red O staining.

### 2.8 Real‐Time Polymerase Chain Reaction (RT‐PCR)

For the in vitro experiments, RT‐PCR was used to detect the relative gene expression levels in hADSCs post‐modRNA transfection and RAW264.7 cells treated with CM (culture media), LPS (lipopolysaccharide, 100 ng/mL, 24 h), LPS + ADSC‐CM, LPS + ADSC^modGFP^‐CM, and LPS + ADSC^modIL‐10^‐CM. For the in vivo experiment, RT‐PCR was used to detect the relative gene expression levels in wound tissues harvested on Day 7 post‐wounding. The total RNA of cells and tissues was extracted by using an RNA‐Quick Purification Kit (Yishan, RN001) and an RNeasy Mini kit (Qiagen, Inc., Valencia, CA), respectively. The RNA concentration was evaluated by optical density at 260 nm using a spectrophotometer. The reverse transcription reaction was carried out using a reverse transcription kit (Vazyme, Nanjing, China) from 1 μg RNA. The reverse transcription reaction was carried out using PrimeScript™ RT Master Mix (Takara, RR036A). RT‐PCR was performed using TB Green® Premix Ex Taq™ (Takara, RR420A) and detected by a LightCycler 480 II real‐time PCR system (Roche, USA). Finally, we determined the relative gene expression levels by using the 2^−(△△CT)^ method and normalized to GAPDH. All primer sequences used in this study were provided in the supporting information (Table S2).

### 2.9 Flow Cytometry

After seeding the RAW264.7 cells onto the plate, stimulate cells for 24 h with untreated‐CM, LPS, LPS + ADSC‐CM, LPS + ADSC^modGFP^‐CM, and LPS + ADSC^modIL‐10^‐CM. Then harvest the macrophages in all groups and prepare them for flow cytometry analysis. Detach the cells using gentle mechanical methods and wash them with an appropriate buffer. The cells were incubated with CCR7/CD197 antibody (BioLegend, 120,107) and CD206 antibody (BioLegend, 141,719) at 4°C for 1 h in a dark place. The cells were then washed in PBS and detected using flow cytometry (BD FACSCelesta, USA). The results were analyzed using FlowJo software (FlowJo LLC, Ashland, OR, USA).

### 2.10 Cell Immunofluorescence Staining

After seeding the RAW264.7 cells onto the plate, stimulate cells for 24 h with untreated‐CM, LPS, LPS + ADSC‐CM, LPS + ADSC^modGFP^‐CM, and LPS + ADSC^modIL‐10^‐CM. Following stimulation, the culture medium was removed and the RAW264.7 cells were fixed with 4% paraformaldehyde for 15–30 min at RT. Subsequently, the cells were permeabilized using 0.1% Triton X‐100 in PBS for 5–10 min and blocked with a blocking buffer for 1 h at RT. The RAW264.7 cells were then incubated overnight at 4°C with specific primary antibodies (iNOS or Arginase‐1, Abcam). Afterward, the cells were incubated with Alexa Fluor 594 secondary antibodies (Abcam) for 1 h at RT. The nuclei were stained with DAPI (blue) for 5–10 min at RT. A confocal laser scanning microscope (Leica) was used to visualize the stained macrophages. ImageJ analysis software was employed to analyze the images and quantify the proportions of M1 and M2 macrophages based on the expression of specific markers.

### 2.11 Skin Wound Healing Assay in Diabetic Murine Model

Male db/db mice (BKS‐Leprem2Cd479/Gpt, Strain NO. T002407, 8 weeks) were purchased from GemPharmatech and acclimated to the animal facility (~20°C, ~45% humidity, and 12/12 light/dark cycle) for one week before the experiment. To minimize individual variances and potential interactions among the animals, we specifically selected rats of the same sex and closely matched body weights for our experiments. The study protocol was approved by the Animal Ethics Committee of Shanghai Yishang Biotechnology Co., Ltd (YS‐JL‐001). Animal experiments were conducted in accordance with the Guide for the Care and Use of Laboratory Animals (US National Institutes of Health, Bethesda, MD, United States). Regular chow and water were available ad libitum. A total of 30 db/db mice (blood glucose >500 mg/dL) were randomly divided into three groups (ten mice per group) using a random digit table. Mice were weighed and anesthetized using tribromoethanol. Two 5‐mm full‐thickness wounds were made using a circular biopsy‐punch (KAI Medical) at the same level on the depilated and disinfected dorsum on either side of the midline according to established methods.^27^, ^38^, ^39^ PBS, 2.5 × 10^5^ hADSCs, or 2.5 × 10^5^ IL‐10 modRNA‐transfected hADSCs (hADSCs^modIL‐10^) were directly injected around the wound. For the same mouse, treatment on Day 0 was applied on the left‐side wound, and treatment on Day 5 was applied on the right‐side wound. A donut‐shaped silicone splint with a 6‐mm/8‐mm diameter was centered around the wound, affixed to the skin using adhesive (Krazy Glue), and interrupted using 6–0 nylon sutures (Ethicon, Somerville, NJ). We also used HyStem®‐HP hydrogels (ESI BIO, Alameda, CA) to create a hydrogel embedding over the wound surface in each group to potentially enhance wound healing synergistically. According to the manufacturer protocol, degassed water‐dissolved Heprasil®, Gelin‐S®, and Extralink® were mixed at a 2:2:1 ratio and used to resuspend a pellet of hADSCs or hADSCs^modIL‐10^ to a concentration of 2.5 × 10^4^ cells/μl within 10 min before treatment. 10 μL of the hydrogel solution containing 2.5 × 10^5^ hADSCs was then administrated to the wound. The wounds were covered with Tegaderm (3 M, Maplewood, MN), which was checked every other day and replaced every three days. The wounds were imaged and the digital photographs of the wounds were taken on Day 0, Day 7, Day 14, and Day 21 post‐surgery (POD0, POD7, POD14, and POD21). Wound areas were digitally measured relative to the inner area of silicone rings (6 mm) and normalized to the original wound areas to calculate the relative wound size.

### 2.12 Histology

On specific time points (POD7 and POD21), wound tissues were harvested with an 8‐mm punch biopsy tool. The wound tissues of each group harvested on POD7 were divided into two parts along their central axis. One half was fixed with 4% paraformaldehyde (Sigma‐Aldrich), embedded with paraffin (Thermo Scientific Chemicals), and sectioned for immunofluorescence staining. The other half was used for RNA extraction. For immunofluorescence staining, sections were permeabilized with 0.1% (v/v) Triton X‐100 in PBS for 20 min, blocked with 5% (v/v) bovine serum albumin for 30 min, and incubated overnight with primary antibodies against F4/80 (rat IgG, Abcam), and CD206 (mouse IgG, Abcam). The following secondary antibodies were purchased from Invitrogen: anti‐rat IgG‐TRITC and anti‐mouse IgG‐FITC. After counterstaining with 4′,6‐diamidino‐2‐phenylindole (DAPI, Thermo Scientific Chemicals), images were captured with a Leica confocal microscopy and quantified using ImageJ.

Wound tissues of each group harvested on POD21 were fixed using 4% paraformaldehyde (Sigma‐Aldrich), embedded with paraffin (Thermo Scientific Chemicals), and sectioned for Masson's trichrome staining (MTS), hematoxylin and eosin (H&E) staining, and immunohistochemical (IHC) analysis. For immunohistochemical analysis, tissue sections were stained with CD31 antibody (ABclonal) and examined to identify the vascular density. Microvessel density is determined by identifying ‘hotspots’ within tissue sections, where higher concentrations of blood vessels are present. These regions are identified using a light microscope. Microvessels, defined as positively stained endothelial cells or clusters that form vessel‐like structures, are then counted within the hotspots to calculate microvessel density.

### 2.13 Cell labeling and Cell Tracking

IL‐10 modRNA‐transfected hADSCs were labeled with the lipophilic fluorochrome chloromethylbenzamido dialkylcarbocyanine (CM‐Dil, Invitrogen, Thermo Scientific, USA) according to the manufacturer's instruction before injection. Concisely, ADSCs^modIL‐10^ were incubated with CM‐Dil at 37°C for 5 min (1 μg CM‐DiI per 10^6^ cells), followed by a 15‐min incubation at 4°C and subsequent washing twice with sterile PBS for the subsequent treatment as previously described.^40^ On Day 5 and Day 7 after transplantation of hADSCs^modIL‐10^ (PBS as control), wound tissues were excised, cut into 10 μm thick frozen sections, and mounted with 49‐6‐diamidino‐2‐phenylindole (DAPI).^41^ The slides were subsequently imaged and captured under a fluorescence microscope to confirm the survival of hADSCs^modIL‐10^ in vivo.

### 2.14 Statistics and Reproducibility

The data were analyzed using GraphPad Prism 9.0 software. Statistical analysis was performed on pooled data from at least three biologically independent experiments with at least three technical replicates. The significance levels were denoted as *p < 0.05; **p < 0.01; ***p < 0.001; ****p < 0.0001, where *p* < 0.05 indicates statistical significance. The figures display all data points and the analysis procedures are detailed in the figure legends.

## 3 RESULTS

### 3.1 Intense Therapeutic Protein Secretion in IL‐10 ModRNA‐Transfected hADSCs

**FIGURE 1:**
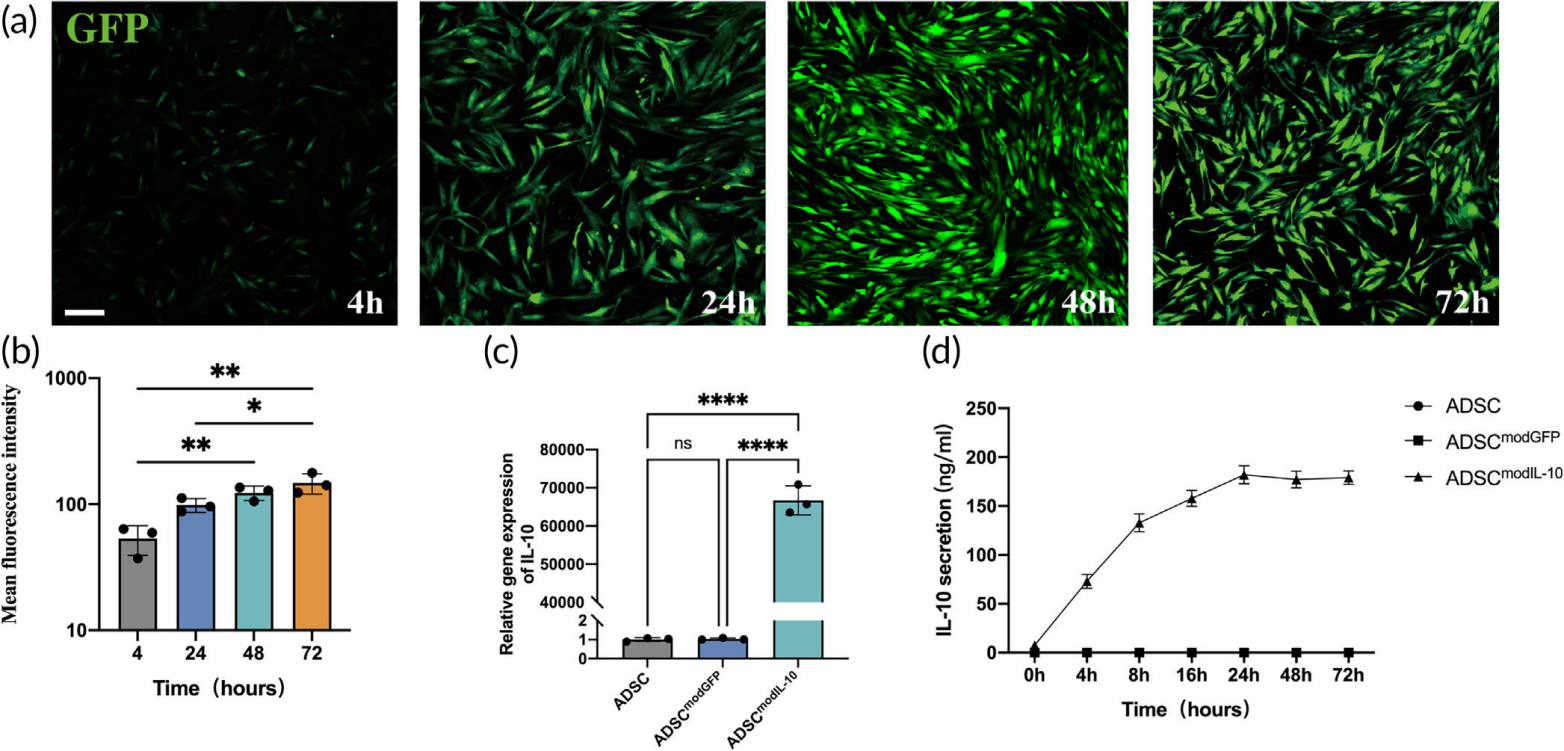
Efficiency and kinetics of modRNA transfection in human adipose‐derived multipotent stromal cells (hADSCs). (a) Representative images of GFP signals in hADSCs^modGFP^ at different time points (4‐, 24‐, 48‐, and 72‐h) after being transfected with GFP modRNA. (b) The histogram presents the analysis of mean fluorescence intensity (Mean = integrated density/area, ImageJ) at 4, 24, 48, and 72 h following transfection. (c) RT‐PCR result shows the relative gene expression of IL‐10 in the untreated ADSC group, the GFP modRNA‐transfected ADSC group (ADSC^modGFP^), and the IL‐10 modRNA‐transfected ADSC group (ADSC^modIL‐10^). The internal control gene, hGAPDH, was utilized for normalization purposes. (d) The ELISA results illustrate the cumulative secretion of IL‐10 protein in each group at various time points (0, 4, 8, 16, 24, 48, and 72 h) following transfection. Data in 1b–d are from *n* = 3 biologically independent samples with 3 technical replicates. Statistical significance and *p* values are analyzed by one‐way ANOVA followed by Tukey's multiple comparisons test. *p < 0.05, **p < 0.01, ***p < 0.001, ****p < 0.0001. Scale bars, 200 μm (a). h, homo sapiens.

To visualize the kinetics, we transfected hADSCs with modRNA encoding a green fluorescent protein (GFP) reporter construct and recorded the mean fluorescence intensity change at 4, 24, 48, and 72 h following transfection (Figure 1a). The results showed that hADSCs were highly tolerant of modRNA transfections. The mean fluorescence intensity significantly increased within 72 h after transfection (Figure 1b). To further determine the relative gene expression of IL‐10 in transfected hADSCs, we conducted RT‐PCR and the results showed a more than 60,000‐fold increase of IL‐10 expression in the ADSC^modIL‐10^ group at 24 h after transfection compared with the control ADSCs group and the ADSC^modGFP^ group (Figure 1c). We further conducted ELISA to detect the accumulated IL‐10 protein levels in the culture media following transfection. Results showed that IL‐10 concentrations in the ADSC^modIL‐10^ culture media were significantly higher 4 h after transfection and peaked around 24 h post‐transfection compared to other groups which exhibited nearly no baseline secretion of IL‐10 (Figure 1d). IL‐10 concentrations only experienced a marginal decrease over the subsequent two days after peaking. This observation underscores the ongoing secretion of IL‐10, which appears to counteract its degradation within the in vitro environment.

### 3.2 The Characteristics of hADSCs Remain Unchanged Following ModRNA Transfection in vitro

**FIGURE 2:**
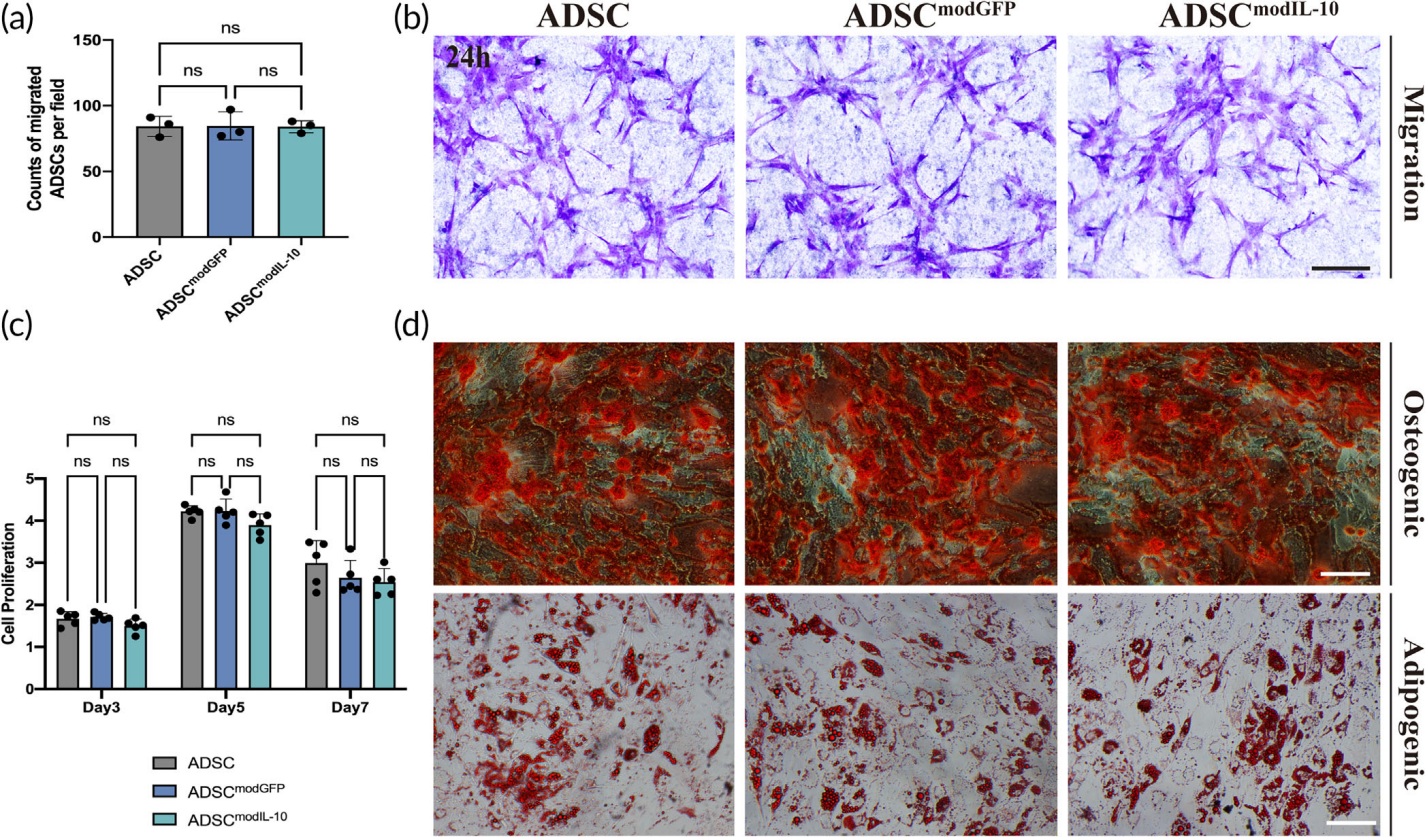
Conservation of hADSCs characteristics in vitro after transfection with modRNA. (a,b) The migratory potentials of untreated ADSCs, ADSCs^modGFP^, and ADSCs^modIL‐10^ were assessed using a Transwell migration assay. The histogram presents the counts of migrated ADSCs per field. Representative images of migrated ADSCs after 24 h, with or without transfection, were displayed. Data in 2a are from *n* = 3 biologically independent samples with 3 technical replicates. Statistical significance and *p* values are analyzed by one‐way ANOVA followed by Tukey's multiple comparisons test. (c) The proliferation capacities of untreated ADSCs, ADSCs^modGFP^, and ADSCs^modIL‐10^ were evaluated using the CCK‐8 (cell counting kit‐8) method. The percentages of optical density values relative to the control group on Day 0 were calculated to determine the relative proliferation rates. Data in 2c are from *n* = 5 biologically independent samples with 3 technical replicates. Statistical significance and *p* values are analyzed by two‐way ANOVA followed by Tukey's multiple comparisons test. (d) The multipotent capacities of hADSCs following modRNA transfection were assessed by inducing osteogenic and adipogenic differentiations. Successful differentiations of the lineages were detected using Alizarin Red S and Oil Red O staining, respectively. *p < 0.05, **p < 0.01, ***p < 0.001, ****p < 0.0001. Scale bars, 20 μm (b), 50 μm (d).

To better testify whether IL‐10 modRNA transfection could function without harming the innate properties of hADSCs. We evaluated the cell proliferation, migration, and differentiation capacity of the untreated ADSCs, ADSCs^modGFP^, and ADSCs^IL‐10^. The migration assay revealed no difference in migration capability among those groups (Figure 2a,b). The results of the CCK8 (Cell Counting Kit‐8) assay showed no significant change in proliferation capacity between modRNA‐transfected groups and the control group (Figure 2c). A decrease in proliferative capacity was observed among all groups on the 7th day, which is likely attributed to cellular contact inhibition. Furthermore, the results of the multipotent differentiation experiment revealed that modRNA transfection did not negatively alter the osteogenic and adipogenic differentiation capacities of the hADSCs (Figure 2d). Collectively, these results suggest that the transfection process did not significantly alter the innate characteristics of hADSCs, indicating their suitability for further applications.

### 3.3 hADSCs^modIL^ ^‐10^ Regulate Macrophage Plasticity into Repair Mode by Prompting M1‐to‐M2 Polarization in vitro

**FIGURE 3:**
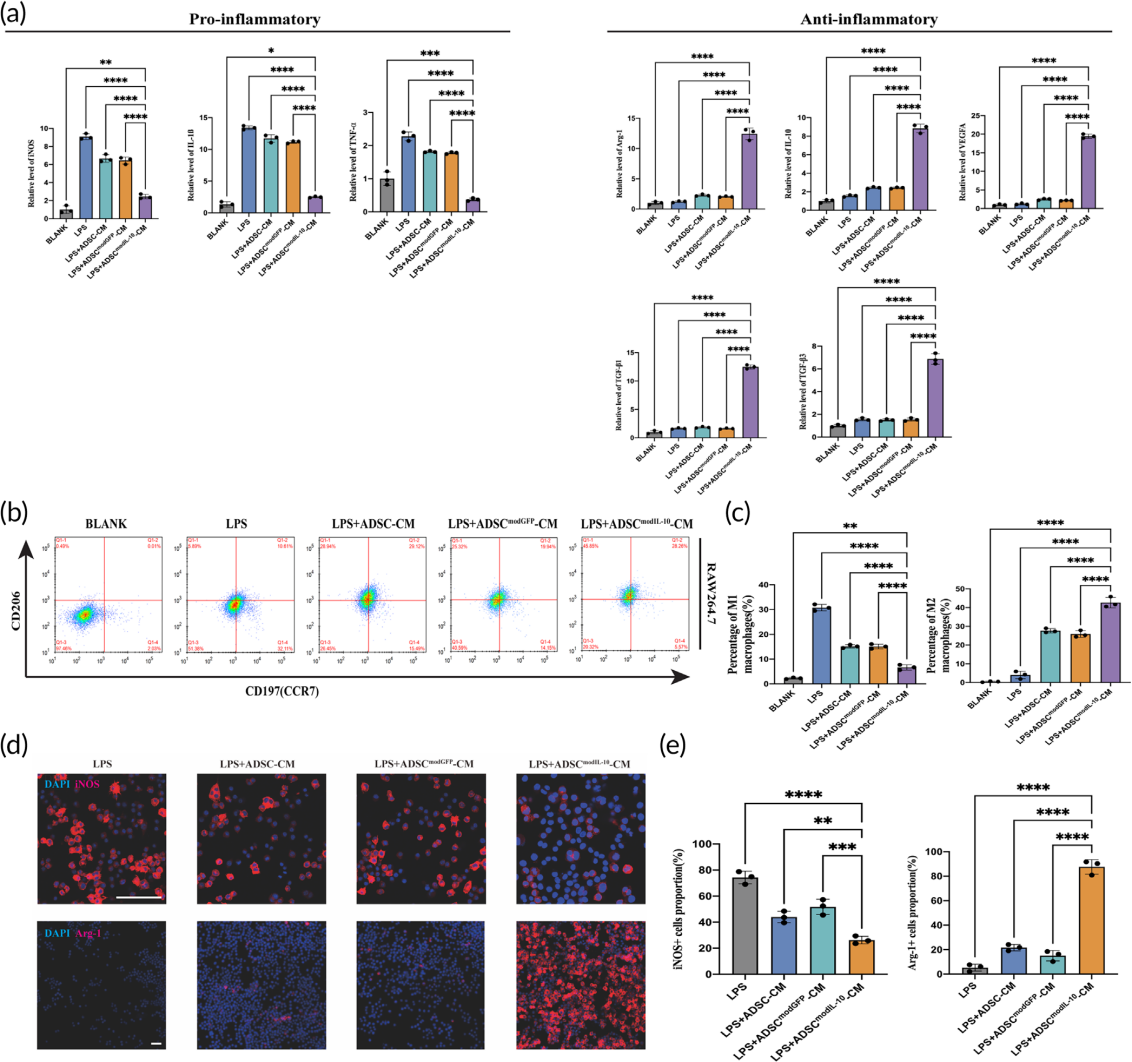
hADSCs^modIL‐10^‐CM promoted anti‐inflammatory macrophage (M2) polarization in vitro. The macrophage cell line RAW264.7 cells were treated with untreated‐CM (BLANK/control), LPS (100 ng/mL), LPS + ADSC‐CM, LPS + ADSC^modGFP^‐CM, and LPS + ADSC^modIL‐10^‐CM for following experiments. (a) The relative levels of M1‐related genes (iNOS, IL‐1β, and TNF‐α) and M2‐related genes (Arg‐1, IL‐10, VEGFA, TGF‐β1, and TGF‐β3) were detected using RT‐PCR. The internal control gene, mGAPDH, was utilized for normalization purposes. Data in 3a are from *n* = 3 biologically independent samples with 3 technical replicates. (b,c) The percentage of M1 macrophages (CD197/CCR7+) and M2 macrophages (CD206+) were detected by flow cytometry assay in each group. (d) Representative images of immunofluorescent staining with DAPI (blue) along with M1 marker iNOS (red) or the M2 marker Arg‐1 (red) in each group. (e) The quantitative analysis of immunofluorescent staining results shows the proportion of iNOS (M1 marker)‐positive cells and Arg‐1 (M2 marker)‐positive cells in each group. Data in 3c and 3e are from *n* = 3 biologically independent samples with 3 technical replicates. Statistical significance and P values are analyzed by one‐way ANOVA followed by Tukey's multiple comparisons test. Only the comparisons to the main treatment group (LPS + ADSC^modIL‐10^‐CM) are shown. *p < 0.05, **p < 0.01, ***p < 0.001, ****p < 0.0001. Scale bars, 50 μm (d). m, Mus musculus.

After treatment with equivalent untreated‐CM, LPS, LPS + ADSC‐CM, LPS + ADSC^modGFP^‐CM, and LPS + ADSC^modIL‐10^‐CM, the relative gene expression levels of the RAW264.7 cells in each group were detected after 24 h of incubation. Results showed that the relative expression levels of pro‐inflammatory M1 macrophage‐related genes (iNOS, IL‐1β, and TNF‐α) were significantly downregulated, while the anti‐inflammatory M2 macrophage‐related genes (Arg‐1, IL‐10, VEGFA, TGF‐β1, and TGF‐β3) were upregulated in the ADSC ^modIL‐10^‐CM treated group (Figure 3a). The macrophage polarization was then evaluated by flow cytometry analysis and immunofluorescence staining in each group. The results of flow cytometry analysis showed that the percentage of M1 macrophage (CCR7/CD197+ cell) was significantly decreased and the percentage of M2 macrophage (CD206+ cell)was significantly increased in the ADSCs^modIL‐10^ group compared with the other groups (Figure 3b,c). The results of IF indicated that the proportion of M1 macrophages (labeled with iNOS, red) was significantly decreased and the proportion of M2 macrophages (labeled with Arg‐1, red) was significantly increased in the ADSC^modIL‐10^‐CM group compared to the other groups (Figure 3d,e). The aforementioned results collectively provide substantial evidence indicating that macrophage plasticity can be effectively manipulated by introducing ADSC^modIL‐10^ into the microenvironment which ultimately facilitates anti‐inflammatory regulation.

### 3.4 Local Administration of hADSCs^modIL^ ^‐10^ in Early Stages of Wound Repair Accelerates Wound Healing in Diabetic Murine Model

**FIGURE 4:**
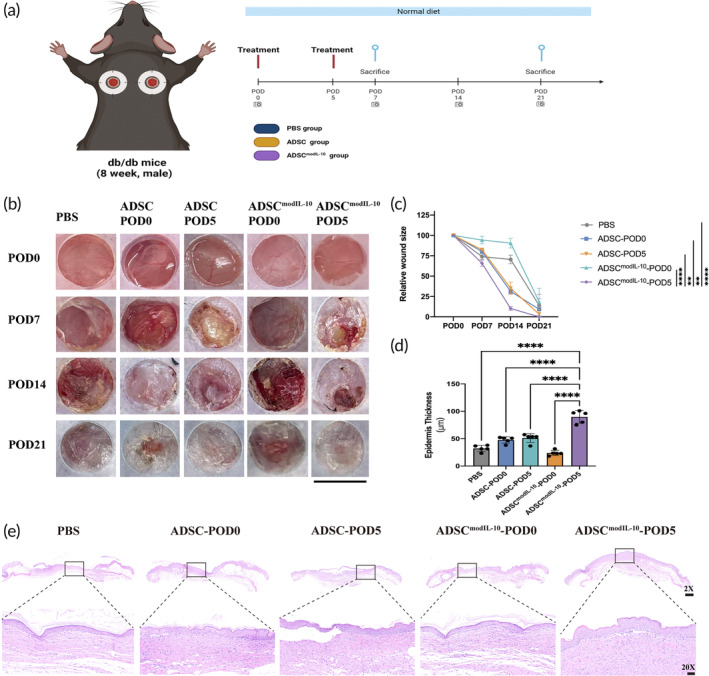
Transplantation of hADSCs^modIL‐10^ on Day 5 post‐wounding accelerated wound healing in diabetic mice. (a) The schematic diagram illustrates the design of the research. Excisional stented wounds were generated on the dorsal skin of hyperglycemic db/db mice. The wounds were then subjected to different treatments, including the administration of PBS or 5 × 10^5^ cells. The cell treatment involved a combination of direct injection of 2.5 × 10^5^ cells and encapsulation of 2.5 × 10^5^ cells within hydrogels. These treatments were conducted either on postoperative days 0 (POD0) or POD5. (b) Representative digital images of the wound area on POD0, 7, 14, and 21, showing the progress of the initial wound closure after injury in each group. (c) The quantification of relative wound size in PBS controls, ADSC‐POD0 (administration of ADSC on POD0), ADSC‐POD5 (administration of ADSC on POD5), ADSC^modIL‐10^‐POD0 (administration of ADSC^modIL‐10^ on POD0), and ADSC^modIL‐10^‐POD5 group (administration of ADSC^modIL‐10^ on POD5). The relative wound size was calculated as the percentage of the current wound size compared to the initial wound size. Data in 4c are from *n* = 5 biologically independent samples. Statistical significance and *p* values are analyzed by two‐way ANOVA followed by Tukey's multiple comparisons test. Only the comparisons to the main treatment group (ADSC^modIL‐10^‐POD5) are shown. (d) Quantification of hematoxylin and eosin (H&E) staining results by calculating the epidermis thickness in the wound area on POD21 in each group. The measurement of epidermal thickness refers to the thickness from the upper edge of the regenerated epidermis to the junction between the epidermis and the dermis at the wound site. Data in 4d are from *n* = 5 biologically independent samples. Statistical significance and *p* values are analyzed by one‐way ANOVA followed by Tukey's multiple comparisons test. Only the comparisons to the main treatment group (ADSC^modIL‐10^‐POD5) are shown. (e) Representative images of H&E staining of wounds harvested on POD21 in each group. *p < 0.05, **p < 0.01, ***p < 0.001, ****p < 0.0001. Scale bars, 6 mm (b), 500 μm (E‐2X), 50 μm (E‐20X).

A schematic of the animal experimental design is shown in Figure 4a and details are summarized in the *MATERIALS & METHODS* section. Wound images were captured at 7‐day intervals until Day 21 (Figure 4b). Transplantation of ADSC^modIL‐10^ on POD5 (ADSC^modIL‐10^‐POD5 group) significantly reduced relative wound size with the most efficient wound‐healing kinetics. Among all groups, the ADSC^modIL‐10^‐POD0 group exhibited the most aberrant healing process (Figure 4b,c). We then used H&E staining to assess the re‐epithelization. The results demonstrated that the administration of IL‐10 during the transition from the inflammation phase to the wound repair phase, as observed in the ADSC^modIL‐10^‐POD5 group, significantly enhanced re‐epithelialization, resulting in the formation of a well‐developed epidermis and thick collagen deposition (Figure 4d,e). Both the PBS and ADSC^modIL‐10^‐POD0 groups exhibited poor wound healing, characterized by thin epidermal thickness and loose dermal collagen (Figure 4e). Collectively, these results demonstrate that transplantation of hADSCs^modIL‐10^ on POD5 could effectively accelerate the wound‐healing process in a diabetic mice model.

### 3.5 Restoration of Aberrant Macrophage Polarization and Regenerative Process Achieved by hADSC^modIL^ ^‐10^ transplanted on Day 5 Post‐Wound

**FIGURE 5:**
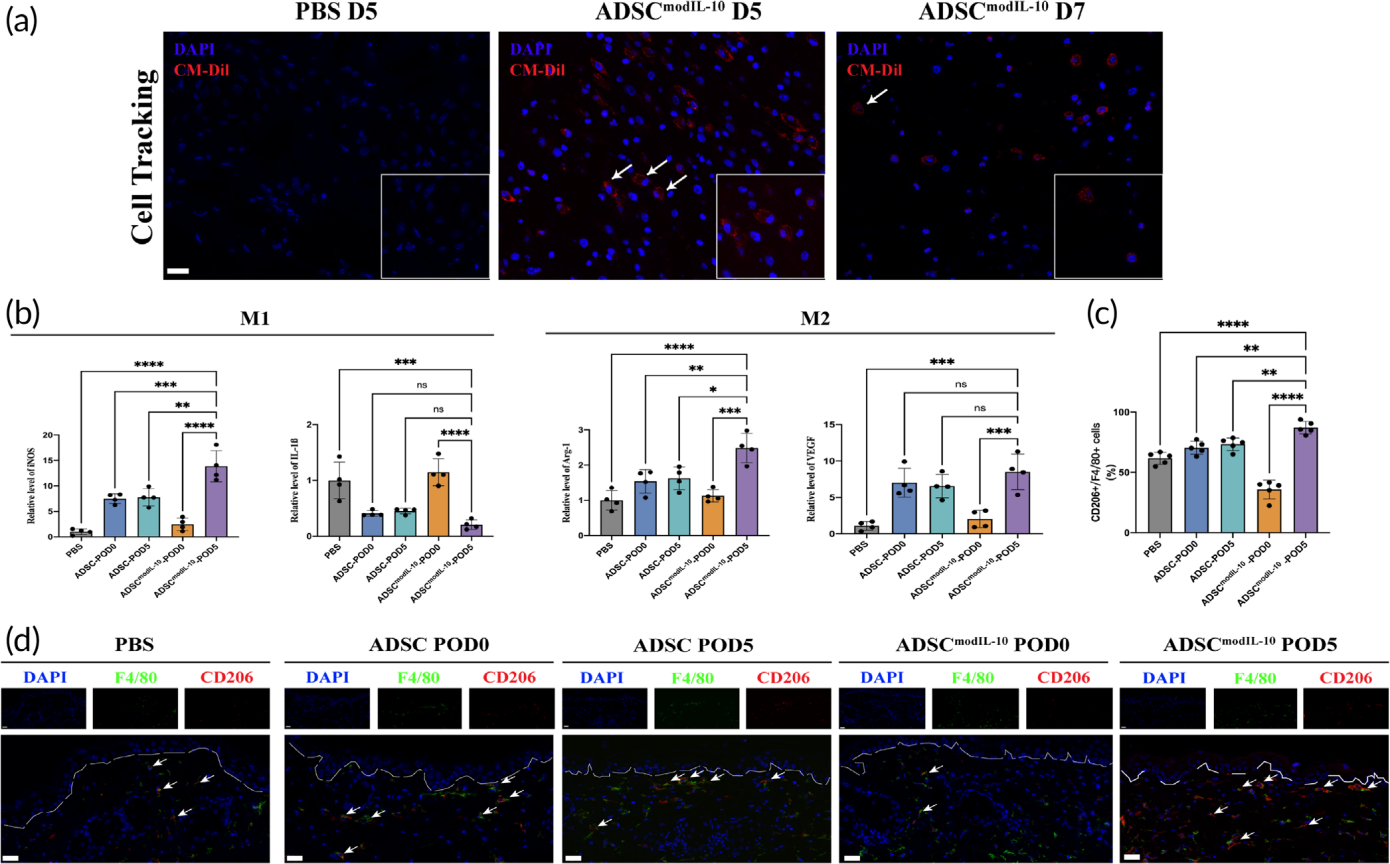
Locally transplanted hADSCs^IL‐10^ survive and promote M2 polarization in diabetic mice. (a) Newly formed wounds in db/db mice were treated with PBS or labeled hADSCs^modIL‐10^. In vivo trackings of transplanted CM‐Dil labeled (red) hADSCs^modIL‐10^ were carried out on Day 5 and Day 7 after transplantation. (b) Wound tissues were harvested on POD7 to detect the relative levels of macrophage‐related genes using RT‐PCR. Data in 5b are from *n* = 4 biologically independent samples with 5 technical replicates. (c) Quantification of IF staining results shows the percentage of M2 macrophages in each group. Data in 5c are from *n* = 5 biologically independent samples. (d) The effects of PBS, hADSC, or hADSC^modIL‐10^ transplanted on POD0 or POD5 were shown by immunofluorescent staining the wound tissue harvested on POD7 with DAPI (blue), F4/80 (green), and CD206 (red) staining. Dotted lines indicate the boundary between the dermis and the epidermis. The white arrows imply the M2 macrophages (F4/80 + CD206+). Statistical significance and *p* values are analyzed by one‐way ANOVA followed by Tukey's multiple comparisons test. Only the comparisons to the main treatment group (ADSC^modIL‐10^‐POD5) are shown.*p < 0.05, **p < 0.01, ***p < 0.001, ****p < 0.0001. Scale bars, 20 μm (a, and d).

To trace the fate of the transplanted hADSCs^modIL‐10^, we detected the red fluorescence signals in the wound tissues harvested at day 5 and day 7 post‐transplantation of CM‐Dil‐labeled hADSCs^modIL‐10^ (Figure 5a). The rationale behind designing the experiment in this manner is based on previous literature suggesting that transplanted stem cells on db/db wounds typically survive for approximately 7 days.^27^ As predicted, the PBS‐treated wound tissues were devoid of red signals on the day5 post‐transplantation. In the hADSC^modIL‐10^‐treated group, the red fluorescence signal decreased over time but still exhibited retention on days 5 and 7 post‐transplantation. Collectively, we conclude that locally administrated hADSCsmodIL‐10 can survive in the early phase of wound healing for at least 7 days despite the immune‐competency of the xenogenic mice model and diabetic inflammation environment. Furthermore, the decrease in the signals with time indicates that the therapeutic effects of hADSCs might function in a ‘hit‐and‐run’ mechanism instead of replacing the local tissues.

**FIGURE 6:**
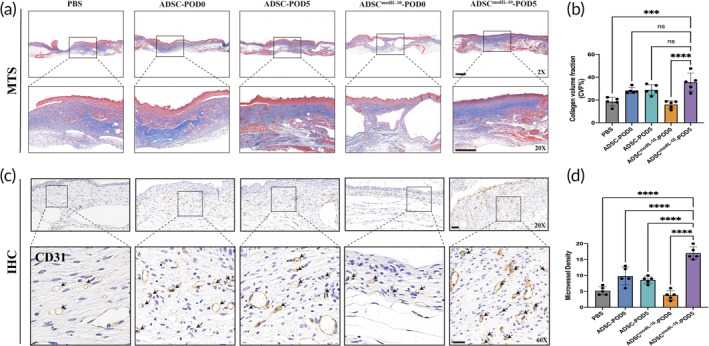
hADSCs^modIL‐10^ transplanted on POD5 enhanced collagen deposition and neovascularization in the regenerative wound area. (a) Representative images of Masson's trichrome staining (MTS) of wound tissues harvested on POD21 in each group. (b) Quantification of MTS results with collagen volume fraction (CVF) to evaluate collagen deposition in each group. (c) Representative images of immunohistochemistry (IHC) staining of CD31 of wound tissues harvested on POD21 in each group (black arrows display neovessels). (d) Quantification of the numbers of neovessels per field as Microvessel Density. Data in 6B and 6D are from *n* = 5 biologically independent samples. Statistical significance and P values are analyzed by one‐way ANOVA followed by Tukey's multiple comparisons test. Only the comparisons to the main treatment group (ADSC^modIL‐10^‐POD5) are shown. *p < 0.05, **p < 0.01, ***p < 0.001, ****p < 0.0001. Scale bars, 500 μm (A‐2X), 300 μm (A‐20X), 50 μm (C‐20X), 20 μm (C‐60X).

The results of tissue RT‐PCR exhibit a beneficial microenvironment in the ADSC^modIL‐10^‐POD5 group, characterized by a decrease in the relative gene expression level of pro‐inflammatory cytokines (iNOS and IL‐1β) and an increase in the relative gene expression level of pro‐healing factors (Arg‐1 and VEGF) (Figure 5b). We then evaluated the in vivo regulation of macrophage phenotypes in wound tissues by co‐staining the POD7 wound sections with monocyte/macrophage marker F4/80 (green) and M2 macrophage marker CD206 (red). Administration of ADSC^modIL‐10^ on POD 5 significantly increased the number of M2 macrophages (F4/80‐positive and CD206‐positive) in diabetic wounds compared with the other groups (Figure 5c,d). Moreover, we observed enhanced collagen fibers and increased collagen volume fraction (CVF) on the POD21 wound in the ADSC^modIL‐10^‐POD5 group compared with the control group (Figure 6a,b). To evaluate the wound bed neovascularization, we used immunohistochemistry (IHC) staining of CD31 to quantify blood vessel density. The ADSC^modIL‐10^‐POD5 group yielded the most significant increase in neovascularization over the PBS, ADSC (‐POD0 and ‐POD5), and ADSC^modIL‐10^‐POD0 group (Figure 6c,d). These data further demonstrate that local delivery of ADSCs^modIL‐10^ on Day 5 post‐wounding may promote the overall healing process by modulating macrophage phenotype shift, promoting collagen synthesis, and neovascularization in diabetic wounds.

## 4 DISCUSSION

Adipose‐derived multipotent stromal cells obtained from fat grafts or liposuctions using various processing procedures^42^ have garnered considerable attention in the field of diabetic wound management^43^, ^44^ and other clinical applications.^45^, ^46^ To enhance their therapeutic effectiveness, it is necessary to explore avenues to modify ADSCs, such as gene editing, nanotechnology, and biomaterial‐assisted.^47^, ^48^, ^49^ While we have previously shown that modified mRNA‐assisted ADSC therapy can ingeniously integrate classical cytokine and stem cell therapies to promote scar‐less healing,^35^ to the best of our knowledge, the utilization of an ADSC‐mediated IL‐10 modRNA delivery system for promoting diabetic wound healing has not been documented before. This underscores the novelty and potentially groundbreaking nature of our research, as we aim to investigate the potential of IL‐10 modRNA‐transfected human ADSCs in modulating the inflammatory response and enhancing regenerative outcomes in a murine diabetic wound model.

To improve the therapeutic protein IL‐10 generating‐ability, we use chemically modified IL‐10 mRNA to transfect the hADSCs. Our research findings are consistent with previous studies, demonstrating that modRNA transfection of hADSCs is highly efficient and allows for rapid and intensive expression of therapeutic proteins.^30^ It only takes hours to secrete a significant amount of proteins, and the cumulative protein concentration can be sustained for over three days. The duration is adequate for ADSCs to achieve therapeutic effects with a ‘Hit‐and‐run’ mechanism to generate therapeutic proteins without replacing the local tissues. Furthermore, our experimental results confirmed that modRNA transfection does not alter the characteristics of ADSCs, including their proliferation, migration, and multipotent differentiation abilities. ModRNA transfection itself does not involve integration into the genome. It simply utilizes the translational machinery of transfected cells to facilitate protein expression without oncogenic risks. Furthermore, the collected conditioned medium from IL‐10 modRNA‐transfected hADSCs was utilized for macrophage intervention. The results revealed that the culture medium, enriched with accumulated IL‐10, notably facilitated anti‐inflammatory M2 macrophage polarization. These findings provide additional confirmation that transfection enables hADSCs to express a significant quantity of therapeutic proteins, thus enhancing their potential application in the management of diabetic wounds.

We further conducted an in vivo study in an excisional wound model in diabetic db/db mice to confirm that IL‐10 modRNA transfected‐hADSCs can carry out their functions in the local wound tissues.^50^ Unlike wild‐type rodents, genetically diabetic mice (db/db mice) with a mutation in the leptin receptor gene exhibit a healing process primarily driven by re‐epithelialization rather than contraction, resembling the wound healing process observed in humans. This characteristic makes them an ideal model for investigating delayed diabetic wound repair, with potential implications for patients with diabetic foot ulcers (DFU).^39^, ^51^ Additionally, we sought to determine the impact of the timing of IL‐10 administration on the therapeutic outcomes. As mentioned earlier, the 5 to 10‐day period following the injury is a critical phase for macrophage phenotype shift and serves as an intervention point for diabetic wound management. Following the previous studies targeting the same time frame,^13^ we selected day 5 post‐operation (POD5) as the starting point for treatment accordingly. Additionally, numerous studies have indicated that overexpression of IL‐10 in the early stages of wound healing is not a favorable signal. Hence, we also chose to deliver ADSC^modIL‐10^ on postoperative day 0 to investigate the impact of the timing of IL‐10 treatment on the outcomes.

Compared to non‐diabetic mice which generally take approximately 9 days to heal based on previous research,^52^ the wounds in the PBS‐treated control diabetic mice in our research remained unhealed even after 14 days. Overall, our findings demonstrate that local transplantation of hADSCs^modIL‐10^ on POD5 significantly promotes diabetic wound healing. This intervention resulted in an accelerated wound closure with better epidermis regeneration, increased anti‐inflammatory M2 macrophage polarization, enhanced collagen deposition, and improved neovascularization in the murine diabetic model. It is worth noting that increased collagen deposition in diabetic wounds is not necessarily a negative outcome. Compared to normal wounds, diabetic wounds exhibit thinner and sparser collagen deposition.^53^ Both diabetic animals and humans have been reported to exhibit decreased levels of TGF‐β in the wound tissue, which is responsible for the delayed wound healing process.^54^, ^55^ However, it is important to note that further research utilizing longer‐term studies in large animal models that closely resemble human wounds is required to thoroughly analyze the fibrosis, since the increased collagen deposition and TGF‐β were observed in our current studies.

Interestingly, contrasting results were observed when hADSCs^modIL‐10^ was administered in the very early phase of wounding (POD0). Those results highlight the critical role of timing in the administration of hADSCs^modIL‐10^ for diabetic wound healing management. Generally, IL‐10 is regarded as a favorable factor that is inadequate in diabetic wounds. Paradoxically, during the acute phase early after injury, inadequate inflammatory response with increased IL‐10 expression in diabetic wounds is proved to further dampen the wound‐healing process.^56^, ^57^ This has also been supported by the clinical finding that elevated pro‐inflammatory characteristics (such as M1 to M2 ratio and enhanced NF‐kB signaling) early after injury was a favorable prognostic marker for healing DFUs.^58^ Hence, IL‐10 is now being widely studied for its biphasic and opposite expression pattern in diabetic wounds.^14^ Previous research showed that initial exposure to high glucose (HG) drove monocytes to increase the expression and secretion level of IL‐10 which later shifted to shut down of IL‐10 due to prolonged cellular damage caused by HG. This might explain the early incompetence of diabetic wounds to start inflammation. Our data showed a similar trend, suggesting that an early burst production of IL‐10 in diabetic wounds is not a favorable indicator and can lead to a worsened wound‐healing outcome. Thus, local transplantation of hADSCs^modIL‐10^ during the macrophage phenotype transition phase (around POD5) serves as a potent therapeutic approach to accelerate diabetic wound healing without disrupting the early inflammatory response.

More preclinical research should be carried out to narrow the specific time window of hADSCs^modIL‐10^ transplantation and determine the optimal dosage of IL‐10 modRNA‐enriched hADSCs for administration, ensuring that it could be safely manufactured and delivered to patients. Moreover, the final observation time point of this experiment was 21 days after wound formation, which corresponds to the time point at which most experimental groups exhibited wound healing. We designed the study in this manner based on the consideration that many clinical trial studies suggest that local transplanted ADSCs do not persist long‐term in the recipient sites and are more likely to exert their effects in a ‘hit‐and‐run’ mechanism rather than retain and replace the host tissue.^22^, ^23^ Relevant research results also support the survival of engineered human ADSCs transplanted onto db/db mouse wounds for approximately 7–9 days.^27^ Therefore, in this study, we did not include longer‐term observation time points. However, longer‐term observations are crucial to comprehensively understand the therapeutic potential and safety profile of a novel treatment.

## 5 CONCLUSION

In conclusion, while our study revealed that IL‐10 modRNA‐enriched hADSCs hold great promise for diabetic wound management, caution must be exercised in the timing of transplantation. Our findings emphasize the need for meticulous consideration while selecting the optimal treatment window for IL‐10‐enhanced cell‐based therapies in diabetic wound healing. By continuing to refine this approach, we can develop more targeted and effective treatments to address the persistent challenge of impaired wound healing in diabetic patients.

## AUTHOR CONTRIBUTIONS

**Yuxin Zhang:** Conceptualization; data curation; formal analysis; investigation; methodology; software; visualization; writing – original draft; writing – review and editing. **Wei Wang:** Data curation; formal analysis; investigation; methodology; software; visualization; writing – original draft; writing – review and editing. **Liang Chen:** Data curation; formal analysis; investigation; software; visualization; writing – original draft; writing – review and editing. **Heng Wang:** Investigation; software; visualization. **Dong Dong:** Formal analysis; methodology; writing – original draft. **Jingjing Zhu:** Data curation; supervision; visualization. **Yu Guo:** Formal analysis; validation; visualization. **Yiqun Zhou:** Data curation; resources; software. **Wei Fu:** Conceptualization; funding acquisition; project administration; resources; supervision; writing – original draft; writing – review and editing. **Tianyi Liu:** Conceptualization; funding acquisition; project administration; resources; supervision; writing – original draft; writing – review and editing.

## CONFLICT OF INTEREST STATEMENT

The authors declare no competing interests.

## Supporting information


**FIGURE S1.** Flow cytometry analysis was performed to examine the surface immunophenotypes of adipose‐derived stem cells (ADSCs). The expression levels of CD73 (a), CD90 (b), CD105 (c), and CD45 (d) were evaluated. The blank control is represented by blue peaks, while the marked ADSCs are represented by red peaks.

**TABLE S1.** Coding sequences for human IL‐10 and GFP modRNA.

**TABLE S2.** Quantitative real‐time PCR primers used in this study.

## Data Availability

The datasets used and/or analyzed during the current study are available from the corresponding author on a reasonable request.

## References

[1] International Diabetes Federation , ed. IDF Diabetes Atlas. 10th ed. International Diabetes Federation; 2021.

[2] Sen CK , Gordillo GM , Roy S , et al. Human skin wounds: a major and snowballing threat to public health and the economy. Wound Repair Regen. 2009;17(6):763‐771. 19903300 10.1111/j.1524-475X.2009.00543.x PMC2810192

[3] Armstrong DG , Swerdlow MA , Armstrong AA , Conte MS , Padula WV , Bus SA . Five year mortality and direct costs of care for people with diabetic foot complications are comparable to cancer. J Foot Ankle Res. 2020;13(1):16. 32209136 10.1186/s13047-020-00383-2 PMC7092527

[4] Aitcheson SM , Frentiu FD , Hurn SE , Edwards K , Murray RZ . Skin wound healing: Normal macrophage function and macrophage dysfunction in diabetic wounds. Molecules. 2021;26(16):4917. 10.3390/molecules26164917 PMC8398285 34443506

[5] Barman PK , Urao N , Koh TJ . Diabetes induces myeloid bias in bone marrow progenitors associated with enhanced wound macrophage accumulation and impaired healing. J Pathol. 2019;249(4):435‐446. 31342513 10.1002/path.5330 PMC8212828

[6] Gunassekaran GR , Poongkavithai Vadevoo SM , Baek MC , Lee B . M1 macrophage exosomes engineered to foster M1 polarization and target the IL‐4 receptor inhibit tumor growth by reprogramming tumor‐associated macrophages into M1‐like macrophages. Biomaterials. 2021;278:121137. 34560422 10.1016/j.biomaterials.2021.121137

[7] Shapouri‐Moghaddam A , Mohammadian S , Vazini H , et al. Macrophage plasticity, polarization, and function in health and disease. J Cell Physiol. 2018;233(9):6425‐6440. 10.1002/jcp.26429 PMC13482560 29319160

[8] Ye Y , Xu Y , Lai Y , et al. Long non‐coding RNA cox‐2 prevents immune evasion and metastasis of hepatocellular carcinoma by altering M1/M2 macrophage polarization. J Cell Biochem. 2018;119(3):2951‐2963. 29131381 10.1002/jcb.26509

[9] Zhu D , Johnson TK , Wang Y , et al. Macrophage M2 polarization induced by exosomes from adipose‐derived stem cells contributes to the exosomal proangiogenic effect on mouse ischemic hindlimb. Stem Cell Res Ther. 2020;11(1):162. 32321589 10.1186/s13287-020-01669-9 PMC7178595

[10] Miao M , Niu Y , Xie T , Yuan B , Qing C , Lu S . Diabetes‐impaired wound healing and altered macrophage activation: a possible pathophysiologic correlation. Wound Repair Regen. 2012;20(2):203‐213. 22380690 10.1111/j.1524-475X.2012.00772.x

[11] Knipper JA , Ding X , Eming SA . Diabetes impedes the epigenetic switch of macrophages into repair mode. Immunity. 2019;51(2):199‐201. 31433963 10.1016/j.immuni.2019.07.009

[12] Mirza R , DiPietro LA , Koh TJ . Selective and specific macrophage ablation is detrimental to wound healing in mice. Am J Pathol. 2009;175(6):2454‐2462. 19850888 10.2353/ajpath.2009.090248 PMC2789630

[13] Ouyang W , Rutz S , Crellin NK , Valdez PA , Hymowitz SG . Regulation and functions of the IL‐10 family of cytokines in inflammation and disease. Annu Rev Immunol. 2011;29:71‐109. 21166540 10.1146/annurev-immunol-031210-101312

[14] Ip WKE , Hoshi N , Shouval DS , Snapper S , Medzhitov R . Anti‐inflammatory effect of IL‐10 mediated by metabolic reprogramming of macrophages. Science. 2017;356(6337):513‐519. 28473584 10.1126/science.aal3535 PMC6260791

[15] Roy R , Zayas J , Mohamed MF , et al. IL‐10 dysregulation underlies chemokine insufficiency, delayed macrophage response, and impaired healing in diabetic wounds. J Invest Dermatol. 2022;142(3 Pt A):692‐704.e14. 34517005 10.1016/j.jid.2021.08.428 PMC8860852

[16] Zhang C , Delawary M , Huang P , Korchak JA , Suda K , Zubair AC . IL‐10 mRNA engineered MSCs demonstrate enhanced anti‐inflammation in an acute GvHD model. Cells. 2021;10:11. 10.3390/cells10113101 PMC8621791 34831324

[17] Liao W , Pham V , Liu L , et al. Mesenchymal stem cells engineered to express selectin ligands and IL‐10 exert enhanced therapeutic efficacy in murine experimental autoimmune encephalomyelitis. Biomaterials. 2016;77:87‐97. 26584349 10.1016/j.biomaterials.2015.11.005 PMC4684451

[18] Niu J , Yue W , Song Y , et al. Prevention of acute liver allograft rejection by IL‐10‐engineered mesenchymal stem cells. Clin Exp Immunol. 2014;176(3):473‐484. 24527865 10.1111/cei.12283 PMC4008992

[19] Levy O , Zhao W , Mortensen LJ , et al. mRNA‐engineered mesenchymal stem cells for targeted delivery of interleukin‐10 to sites of inflammation. Blood. 2013;122(14):e23‐e32. 23980067 10.1182/blood-2013-04-495119 PMC3790516

[20] Li X , Zhang G , Wang M , et al. Comparison of stromal vascular fraction cell composition between Coleman fat and extracellular matrix/stromal vascular fraction gel. Adipocyte. 2024;13(1):2360037. 38829527 10.1080/21623945.2024.2360037 PMC11152101

[21] Liu R , Dong R , Chang M , Liang X , Wang HC . Adipose‐derived stem cells for the treatment of diabetic wound: from basic study to clinical application. Front Endocrinol (Lausanne). 2022;13:882469. 35898452 10.3389/fendo.2022.882469 PMC9309392

[22] von Bahr L , Batsis I , Moll G , et al. Analysis of tissues following mesenchymal stromal cell therapy in humans indicates limited long‐term engraftment and no ectopic tissue formation. Stem Cells. 2012;30(7):1575‐1578. 22553154 10.1002/stem.1118

[23] François M , Romieu‐Mourez R , Li M , Galipeau J . Human MSC suppression correlates with cytokine induction of indoleamine 2,3‐dioxygenase and bystander M2 macrophage differentiation. Mol Ther. 2012;20(1):187‐195.21934657 10.1038/mt.2011.189

[24] Kimbrel EA , Lanza R . Next‐generation stem cells ‐ ushering in a new era of cell‐based therapies. Nat Rev Drug Discov. 2020;19(7):463‐479. 32612263 10.1038/s41573-020-0064-x

[25] Levy O , Kuai R , Siren EMJ , et al. Shattering barriers toward clinically meaningful MSC therapies. Sci Adv. 2020;6(30):eaba6884. 32832666 10.1126/sciadv.aba6884 PMC7439491

[26] Wu C , Dunbar CE . Stem cell gene therapy: the risks of insertional mutagenesis and approaches to minimize genotoxicity. Front Med. 2011;5(4):356‐371. 22198747 10.1007/s11684-011-0159-1 PMC3508510

[27] Srifa W , Kosaric N , Amorin A , et al. Cas9‐AAV6‐engineered human mesenchymal stromal cells improved cutaneous wound healing in diabetic mice. Nat Commun. 2020;11(1):2470. 32424320 10.1038/s41467-020-16065-3 PMC7235221

[28] Yu F , Witman N , Yan D , et al. Human adipose‐derived stem cells enriched with VEGF‐modified mRNA promote angiogenesis and long‐term graft survival in a fat graft transplantation model. Stem Cell Res Ther. 2020;11(1):490. 33213517 10.1186/s13287-020-02008-8 PMC7678328

[29] Lui KO , Zangi L , Chien KR . Cardiovascular regenerative therapeutics via synthetic paracrine factor modified mRNA. Stem Cell Res. 2014;13(3 Pt B):693‐704. 25043723 10.1016/j.scr.2014.06.007

[30] Geng Y , Duan H , Xu L , et al. BMP‐2 and VEGF‐A modRNAs in collagen scaffold synergistically drive bone repair through osteogenic and angiogenic pathways. Commun Biol. 2021;4(1):82. 33469143 10.1038/s42003-020-01606-9 PMC7815925

[31] Chien KR , Zangi L , Lui KO . Synthetic chemically modified mRNA (modRNA): toward a new technology platform for cardiovascular biology and medicine. Cold Spring Harb Perspect Med. 2014;5(1):a014035. 25301935 10.1101/cshperspect.a014035 PMC4292072

[32] Yu Z , Witman N , Wang W , et al. Cell‐mediated delivery of VEGF modified mRNA enhances blood vessel regeneration and ameliorates murine critical limb ischemia. J Control Release. 2019;310:103‐114. 31425721 10.1016/j.jconrel.2019.08.014

[33] Pardi N , Hogan MJ , Porter FW , Weissman D . mRNA vaccines ‐ a new era in vaccinology. Nat Rev Drug Discov. 2018;17(4):261‐279. 29326426 10.1038/nrd.2017.243 PMC5906799

[34] Sahin U , Karikó K , Türeci Ö . mRNA‐based therapeutics — developing a new class of drugs. Nat Rev Drug Discov. 2014;13(10):759‐780. 25233993 10.1038/nrd4278

[35] Wang W , Chen L , Zhang Y , et al. Adipose‐derived stem cells enriched with therapeutic mRNA TGF‐β3 and IL‐10 synergistically promote scar‐less wound healing in preclinical models. Bioeng Transl Med. 2024;9(2):e10620. 38435824 10.1002/btm2.10620 PMC10905533

[36] Zhu M , Heydarkhan‐Hagvall S , Hedrick M , Benhaim P , Zuk P . Manual isolation of adipose‐derived stem cells from human lipoaspirates. J vis Exp. 2013;79:e50585. 10.3791/50585 PMC3935774 24121366

[37] Warren L , Manos PD , Ahfeldt T , et al. Highly efficient reprogramming to pluripotency and directed differentiation of human cells with synthetic modified mRNA. Cell Stem Cell. 2010;7(5):618‐630. 20888316 10.1016/j.stem.2010.08.012 PMC3656821

[38] Wang X , Ge J , Tredget EE , Wu Y . The mouse excisional wound splinting model, including applications for stem cell transplantation. Nat Protoc. 2013;8(2):302‐309. 23329003 10.1038/nprot.2013.002

[39] Theocharidis G , Yuk H , Roh H , et al. A strain‐programmed patch for the healing of diabetic wounds. Nat Biomed Eng. 2022;6(10):1118‐1133. 35788686 10.1038/s41551-022-00905-2

[40] Xiong J , Qiang H , Li T , et al. Human adipose‐derived stem cells promote seawater‐immersed wound healing via proangiogenic effects. Aging (Albany NY). 2021;13(13):17118‐17136. 33819183 10.18632/aging.202773 PMC8312430

[41] Zhou L , Song K , Xu L , et al. Protective effects of uncultured adipose‐derived stromal vascular fraction on testicular injury induced by torsion‐Detorsion in rats. Stem Cells Transl Med. 2019;8(4):383‐391. 30569668 10.1002/sctm.18-0063 PMC6431687

[42] Gentile P , Scioli MG , Bielli A , Orlandi A , Cervelli V . Comparing different nanofat procedures on scars: role of the stromal vascular fraction and its clinical implications. Regen Med. 2017;12(8):939‐952. 29236575 10.2217/rme-2017-0076

[43] Hou L , Zhang X , Du H . Advances in mesenchymal stromal cells and nanomaterials for diabetic wound healing. Diabetes Metab Res Rev. 2023;39(4):e3638. 36959689 10.1002/dmrr.3638

[44] Moon KC , Suh HS , Kim KB , et al. Potential of allogeneic adipose‐derived stem cell‐hydrogel complex for treating diabetic foot ulcers. Diabetes. 2019;68(4):837‐846. 30679183 10.2337/db18-0699

[45] Gentile P . New strategies in plastic surgery: autologous adipose‐derived mesenchymal stem cells contained in fat grafting improves symptomatic scars. Front Biosci (Landmark Ed). 2021;26(8):255‐257. 34455756 10.52586/4940

[46] Gentile P , Sterodimas A , Pizzicannella J , et al. Systematic review: allogenic use of stromal vascular fraction (SVF) and Decellularized extracellular matrices (ECM) as advanced therapy medicinal products (ATMP) in tissue regeneration. Int J Mol Sci. 2020;21(14):4982. 32679697 10.3390/ijms21144982 PMC7404290

[47] Eke G , Mangir N , Hasirci N , MacNeil S , Hasirci V . Development of a UV crosslinked biodegradable hydrogel containing adipose derived stem cells to promote vascularization for skin wounds and tissue engineering. Biomaterials. 2017;129:188‐198. 28343005 10.1016/j.biomaterials.2017.03.021

[48] Dong Y , Wu X , Chen X , Zhou P , Xu F , Liang W . Nanotechnology shaping stem cell therapy: recent advances, application, challenges, and future outlook. Biomed Pharmacother. 2021;137:111236. 33486201 10.1016/j.biopha.2021.111236

[49] Xue Y , Che J , Ji X , Li Y , Xie J , Chen X . Recent advances in biomaterial‐boosted adoptive cell therapy. Chem Soc Rev. 2022;51(5):1766‐1794. 35170589 10.1039/d1cs00786f

[50] Dunn L , Prosser HC , Tan JT , Vanags LZ , Ng MK , Bursill CA . Murine model of wound healing. J Vis Exp. 2013;75:e50265. 10.3791/50265 PMC3724564 23748713

[51] Galiano RD , Michaels Jt , Dobryansky M , Levine JP , Gurtner GC . Quantitative and reproducible murine model of excisional wound healing. Wound Repair Regen. 2004;12(4):485‐492. 15260814 10.1111/j.1067-1927.2004.12404.x

[52] Xue Y , Zhang Y , Zhong Y , et al. LNP‐RNA‐engineered adipose stem cells for accelerated diabetic wound healing. Nat Commun. 2024;15(1):739. 38272900 10.1038/s41467-024-45094-5 PMC10811230

[53] Laiva AL , O'Brien FJ , Keogh MB . SDF‐1α gene‐activated collagen scaffold restores pro‐Angiogenic wound healing features in human diabetic adipose‐derived stem cells. Biomedicine. 2021;9:2. 10.3390/biomedicines9020160 PMC7914837 33562165

[54] Patel S , Srivastava S , Singh MR , Singh D . Mechanistic insight into diabetic wounds: pathogenesis, molecular targets and treatment strategies to pace wound healing. Biomed Pharmacother. 2019;112:108615. 30784919 10.1016/j.biopha.2019.108615

[55] Bitar MS , Labbad ZN . Transforming growth factor‐beta and insulin‐like growth factor‐I in relation to diabetes‐induced impairment of wound healing. J Surg Res. 1996;61(1):113‐119. 8769952 10.1006/jsre.1996.0090

[56] Basu Mallik S , Jayashree BS , Shenoy RR . Epigenetic modulation of macrophage polarization‐ perspectives in diabetic wounds. J Diabetes Complications. 2018;32(5):524‐530. 29530315 10.1016/j.jdiacomp.2018.01.015

[57] Ishida Y , Kuninaka Y , Nosaka M , et al. CCL2‐mediated reversal of impaired skin wound healing in diabetic mice by normalization of neovascularization and collagen accumulation. J Invest Dermatol. 2019;139(12):2517‐2527.e5. 31247201 10.1016/j.jid.2019.05.022

[58] Theocharidis G , Baltzis D , Roustit M , et al. Integrated skin Transcriptomics and serum multiplex assays reveal novel mechanisms of wound healing in diabetic foot ulcers. Diabetes. 2020;69(10):2157‐2169. 32763913 10.2337/db20-0188 PMC7506837

