# Supplementary material for: Human adipose‐derived multipotent stromal cells enriched with IL‐10 modRNA improve diabetic wound healing: Trigger the macrophage phenotype shift
Source: Bioeng Transl Med. 2024 Aug 7;10(1):e10711. doi: 10.1002/btm2.10711 (PMC11711206; doi:10.1002/btm2.10711)
Supplement: Supplementary file 1 — FIGURE S1. Flow cytometry analysis was performed to examine the surface immunophenotypes of adipose‐derived stem cells (ADSCs). The expression levels of CD73 (a), CD90 (b), CD105 (c), and CD45 (d) were evaluated. The blank control is represented by blue peaks, while the marked ADSCs are represented by red peaks. [file BTM2-10-e10711-s002.docx]

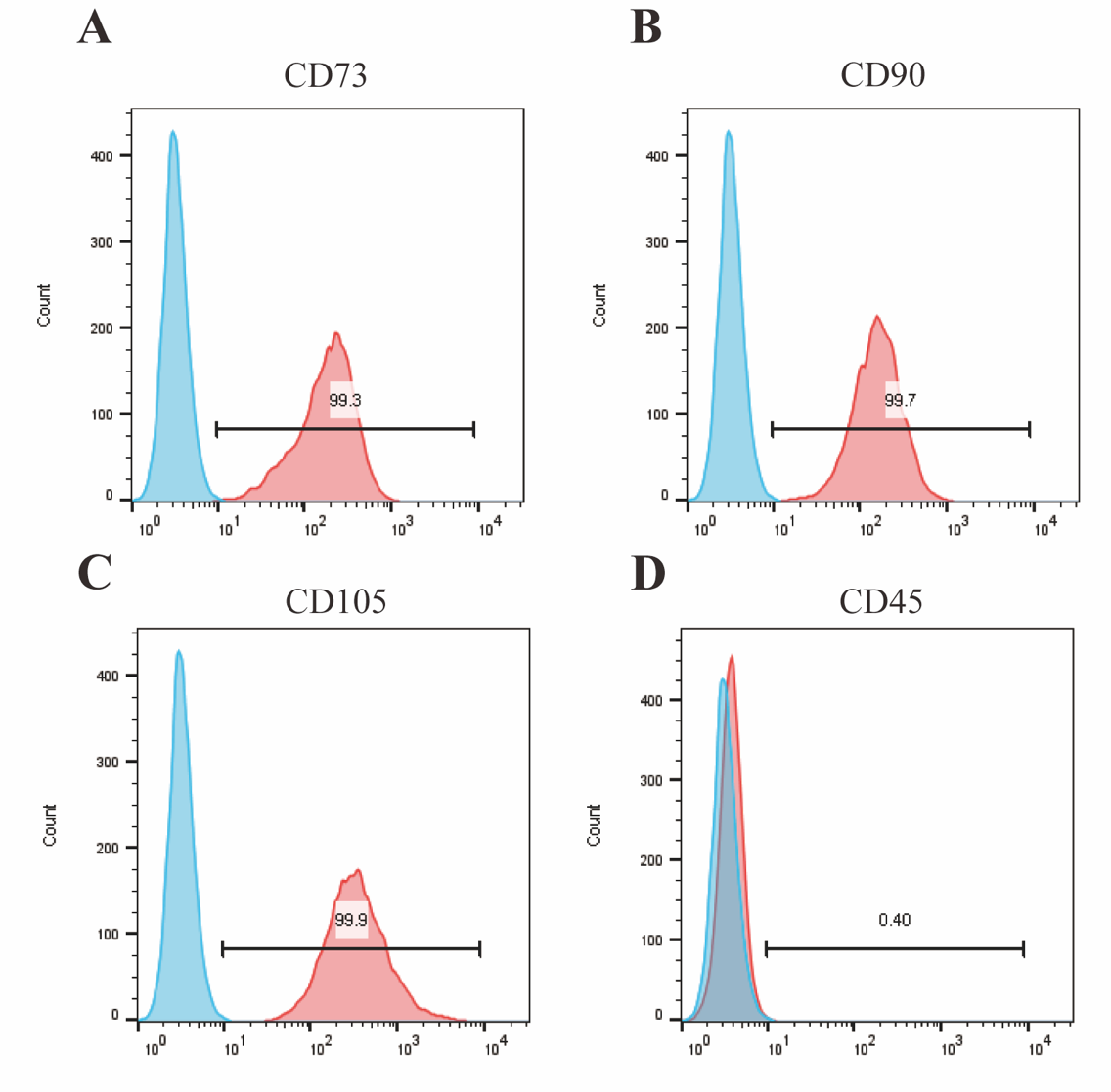


**Figure S1**. Flow cytometry analysis was performed to examine the surface immunophenotypes of adipose-derived stem cells (ADSCs). The expression levels of CD73 (A), CD90 (B), CD105 (C), and CD45 (D) were evaluated. The blank control is represented by blue peaks, while the marked ADSCs are represented by red peaks.
