# Supplementary material for: Human adipose‐derived multipotent stromal cells enriched with IL‐10 modRNA improve diabetic wound healing: Trigger the macrophage phenotype shift
Source: Bioeng Transl Med. 2024 Aug 7;10(1):e10711. doi: 10.1002/btm2.10711 (PMC11711206; doi:10.1002/btm2.10711)
Supplement: Supplementary file 2 — TABLE S1. Coding sequences for human IL‐10 and GFP modRNA. [file BTM2-10-e10711-s003.docx]

**Table S1. Coding sequences for** **human IL-10 and GFP modRNA**

| Human IL-10 CDS | ATGCACAGCTCAGCACTGCTCTGTTGCCTGGTCCTCCTGACTGGGGTGAGGGCCAGCCCAGGCCAGGGCACCCAGTCTGAGAACAGCTGCACCCACTTCCCAGGCAACCTGCCTAACATGCTTCGAGATCTCCGAGATGCCTTCAGCAGAGTGAAGACTTTCTTTCAAATGAAGGATCAGCTGGACAACTTGTTGTTAAAGGAGTCCTTGCTGGAGGACTTTAAGGGTTACCTGGGTTGCCAAGCCTTGTCTGAGATGATCCAGTTTTACCTGGAGGAGGTGATGCCCCAAGCTGAGAACCAAGACCCAGACATCAAGGCGCATGTGAACTCCCTGGGGGAGAACCTGAAGACCCTCAGGCTGAGGCTACGGCGCTGTCATCGATTTCTTCCCTGTGAAAACAAGAGCAAGGCCGTGGAGCAGGTGAAGAATGCCTTTAATAAGCTCCAAGAGAAAGGCATCTACAAAGCCATGAGTGAGTTTGACATCTTCATCAACTACATAGAAGCCTACATGACAATG AAGATACGAAACTGA |
| --- | --- |
| GFP CDS | ATGGTGAGCAAGGGCGAGGAGCTGTTCACCGGGGTGGTGCCCATCCTGGTCGAGCTGGACGGCGACGTAAACGGCCACAAGTTCAGCGTGTCCGGCGAGGGCGAGGGCGATGCCACCTACGGCAAGCTGACCCTGAAGTTCATCTGCACCACCGGCAAGCTGCCCGTGCCCTGGCCCACCCTCGTGACCACCCTGACCTACGGCGTGCAGTGCTTCAGCCGCTACCCCGACCACATGAAGCAGCACGACTTCTTCAAGTCCGCCATGCCCGAAGGCTACGTCCAGGAGCGCACCATCTTCTTCAAGGACGACGGCAACTACAAGACCCGCGCCGAGGTGAAGTTCGAGGGCGACACCCTGGGAACCGCATCGAGCTGAAGGGCATCGACTTCAAGGAGGACGGCAACATCCTGGGGCACAAGCTGGAGTACAACTACAACAGCCACAACGTCTATATCATGGCCGACAAGCAGAAGAACGGCATCAAGGTGAACTTCAAGATCCGCCACAACATCGAGGACGGCAGCGTGCAGCTCGCCGACCACTACCAGCAGAACACCCCCATCGGCGACGGCCCCGTGCTGCTGCCCGACAACCACTACCTGAGCACCCAGTCCGCCCTGAGCAAAGACCCCAACGAGAAGCGCGATCACATGGTCCTGCTGGAGTTCGTGACCGCCGCCGGGATCACTCTCGGCATGGACGAGCTGTACAAGTAA |

IL-10, interleukin-10; GFP, green fluorescent protein
