# Supplementary material for: Human adipose‐derived multipotent stromal cells enriched with IL‐10 modRNA improve diabetic wound healing: Trigger the macrophage phenotype shift
Source: Bioeng Transl Med. 2024 Aug 7;10(1):e10711. doi: 10.1002/btm2.10711 (PMC11711206; doi:10.1002/btm2.10711)
Supplement: Supplementary file 3 — TABLE S2. Quantitative real‐time PCR primers used in this study. [file BTM2-10-e10711-s001.docx]

**Table S2. Quantitative real-time PCR primers used in this study**

| Gene Name | Primer |
| --- | --- |
| h IL-10 | F: AAGACCCAGACATCAAGGCG |
|  | R: AGGCATTCTTCACCTGCTCC |
| m iNOS | F: CTATGGCCGCTTTGATGTGC |
|  | R: TTGGGATGCTCCATGGTCAC |
| m Arg-1 | F: GACAGGGCTCCTTTCAGGAC |
|  | R: CTGTGATGCCCCAGATGGTT |
| m TNF-α | F: GACCCTCACACTCAGATCATCTTCT |
|  | R: CCACTTGGTGGTTTGCTACGA |
| m IL-10 | F: CAGGGCACTAGGTGTTGAGG |
|  | R: GTTCTGATTTGGGGGAGGGG |
| m IL-1β | F: GTCGCTCAGGGTCACAAGAA |
|  | R: CCACACGTTGACAGCTAGGT |
| m VEGFA | F: TTCGAGGAGCACTTTGGGTC |
|  | R: GTGGGTGGGTGTGTCTACAG |
| m TGF-β1 | F: AAACTAAGGCTCGCCAGTCC |
|  | R: CATAGATGGCGTTGTTGCGG |
| m TGF-β3 | F: ATGACCCACGTCCCCTATCA |
|  | R: GACTCCGAGGTCTCCTGAGT |
| m GAPDH | F: CCTCGTCCCGTAGACAAAATG |
|  | R: TGAGGTCAATGAAGGGGTCGT |
| h GAPDH | F: GGGAGCCAAAAGGGTCATCATCTC |
|  | R: GAGGGGCCATCCACAGTCTTC |

h, homo sapiens; m, Mus musculus
